# Could dysbiosis of inflammatory and anti-inflammatory gut bacteria have an implications in the development of type 2 diabetes? A pilot investigation

**DOI:** 10.1186/s13104-021-05466-2

**Published:** 2021-02-06

**Authors:** Prasanna Kulkarni, Poornima Devkumar, Indranil Chattopadhyay

**Affiliations:** 1 Institute of Ayurveda and Integrative Medicine (I-AIM), Bangalore, India; 2 Department of Life Sciences, Central University of Tamil Nadu, Thiruvarur, 610005 India

**Keywords:** Gut microbiota, Type 2 diabetes, Inflammation, Diagnostic marker

## Abstract

**Objective:**

Differential alterations in gut microbiota and chronic low-grade inflammation play a critical role in the development of Type 2 diabetes (T2D). Here we aimed to investigate if dysbiosis of inflammation and anti-inflammation-associated gut bacterial communities in fecal samples of individuals had any influence on T2D using a 16S rRNA gene of V3 region sequencing at Illumina MiSeq platform.

**Results:**

Our findings showed that a higher abundance of inflammatory bacteria such as *Lactobacillus ruminis, Ruminococcus gnavus, Bacteroides caccae, Butyricimonas,* and *Collinsella aerofaciens*, and lower abundance of anti-inflammatory bacteria such as *Faecalibacterium prausnitzii*, and *Butyrivibrio* that likely play a role in the development of T2D. Our findings hint the potential of indigenous microbiota in developing diagnostic markers and therapeutic targets in T2D.

## Introduction

Type 2 diabetes (T2D) is a chronic metabolic disorder characterized by hyperglycemia, insulin resistance, and insufficient secretion of insulin [1]. Studies suggest that nature of gut microbiome determine the onset of T2D through alterations in glucose metabolism and abnormal production of short-chain fatty acids (SCFAs) such acetic acid, propionic acid, and butyric acid [2–4], which appears to hold relevance for the presence or absence of indigenous gut microbiota that appears to correlate with the levels of SCFAs in the host’s gut compartment. The differential levels of gut bacteria holds key to determine the natural history and progression of several human diseases, for instance, tuberculosis, HIV infection, cancer and several autoimmune, hypersensitivity and inflammatory manifestations. Accumulating lines of evidence suggests that lower abundance of butyrate producing bacteria such as *Bifidobacterium, Roseburia, Akkermansia, Blautia*, and *Faecalibacterium prauznitzii* contribute to the outcome of T2D [5–7]. It has also been reported that butyrate induces low-grade inflammation that drives the development of T2DM [8]. Genus *Lactobacillus* is responsible for chronic inflammation in T2D patients [9]. In this study, we hypothesized that dysbiosis of gut bacteria associated with inflammation and anti-inflammatory may have a role in the development of T2D. Significant alterations in the composition of gut microbiota may be considered as potential biomarkers to predict the existence or onset of T2D in individuals. Here, we aimed to examine the dysbiosis of gut microbial communities associated with inflammation in fecal samples of patients with T2D as well as healthy controls.

## Main text

### Materials and methods

Fecal samples were collected from 5 patients with T2D (aged 56 ± 8 years) who were referred to the Institute of Ayurveda and Integrative Medicine (I-AIM), Bangalore, India during July 2017 to December 2017. The inclusion criteria of the current study were a diagnosis of T2D and hemoglobin A1c (HbA1c > 6.5%). The exclusion criteria were as follows: (i) Type 1 and other specific types of diabetes (ii) T2D patients administering oral hypoglycemic drugs /insulin (iii) women who are pregnant, attempting to conceive, or nursing mothers, and/or participants on prolonged therapy (including antibiotics during the last 3 months) (iv) participants with one or more of the following known co-morbid conditions like retinopathy, neuropathy, and nephropathy, participants with acute and chronic gastrointestinal disorders like inflammatory bowel syndrome (IBS), food allergy and lactose intolerance (v) participants with a history of organ transplantation and diseases of the oral cavity. Stool specimens were also collected from 5 healthy individuals without diabetes and with no apparent history of IBS or any GI related problems that warrant long-term medications at least 6 months prior to recruitment in the study, matched for age, gender, and living environment. Healthy individuals recruited in the study had a BMI between18.5 and 25, and did not have a history of antibiotic usage at least 3 months before participation in the investigation.

Fecal samples were collected in a commercial OMNIgene GUT tube (DNA Genotek) as per the manufacturer’s instructions. DNA was extracted from stool samples by using Qiagen DNeasy blood and tissue kit as per manufacturer’s guidelines. Ten nanograms of the genomic DNA was subjected to amplification, and sequencing of the 16S rRNA gene using V3 region-specific primers as per Illumina-recommended primer was performed on an Illumina MiSeq Sequencer (Illumina, Inc.). The Illumina paired end reads (150*2) were demultiplexed using the bcl2fastq tool. The paired end reads were quality checked using FastQC. The raw reads having primer sequence and high quality bases were selected, and the reads were further stitched using Fastq-join. These stitched reads were considered for further analysis using QIIME pipeline. The query sequences were clustered using UCLUST method. The taxonomy of these clusters was assigned based on >  = 97% sequence similarity against the curated chimera free16s rRNA database (Greengenes v 13.8). The functional analysis was done by using Phylogenetic Investigation of Communities by Reconstruction of Unobserved States (PICRUSt) tool.

### Results

**Fig. 1:**
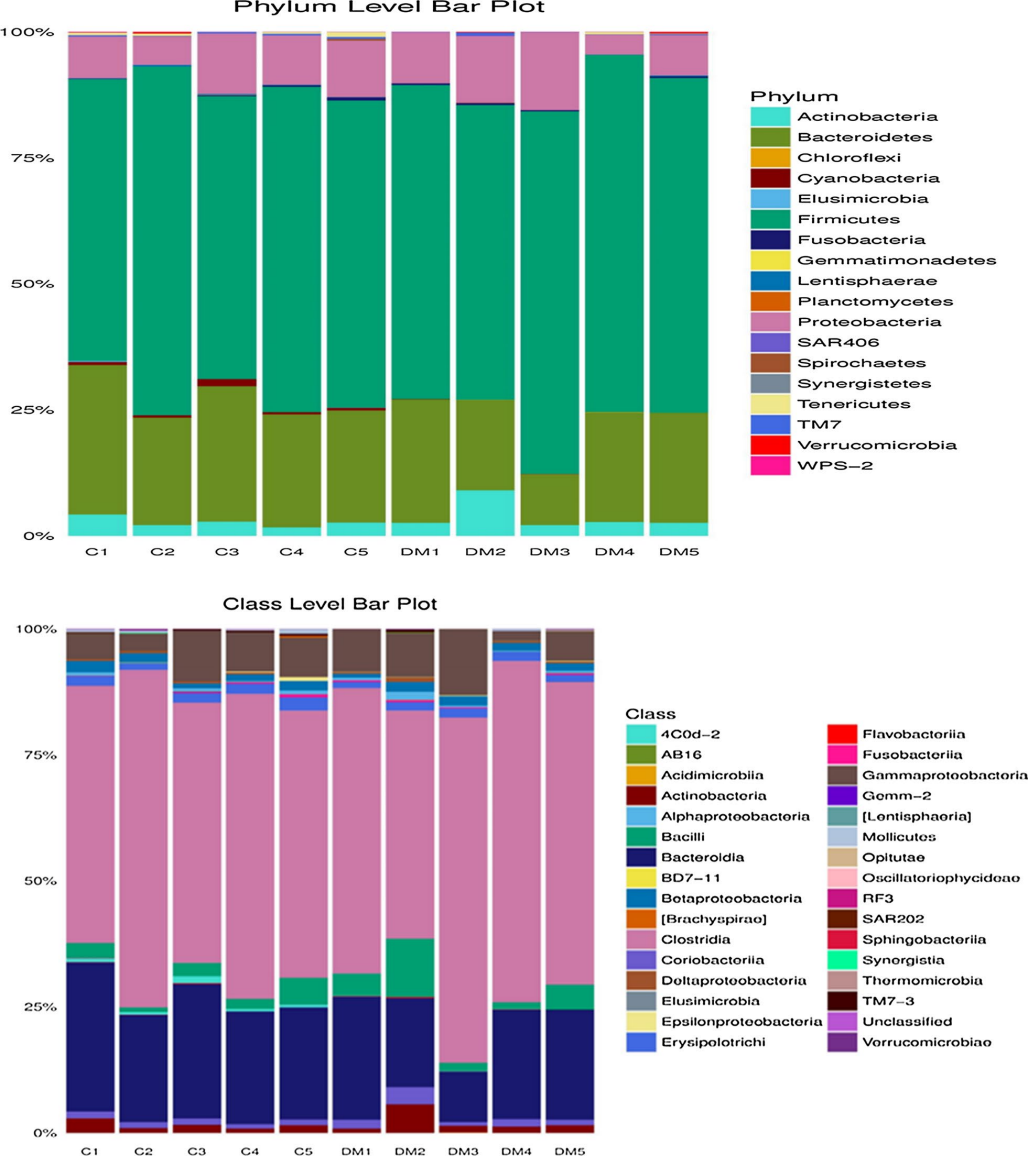

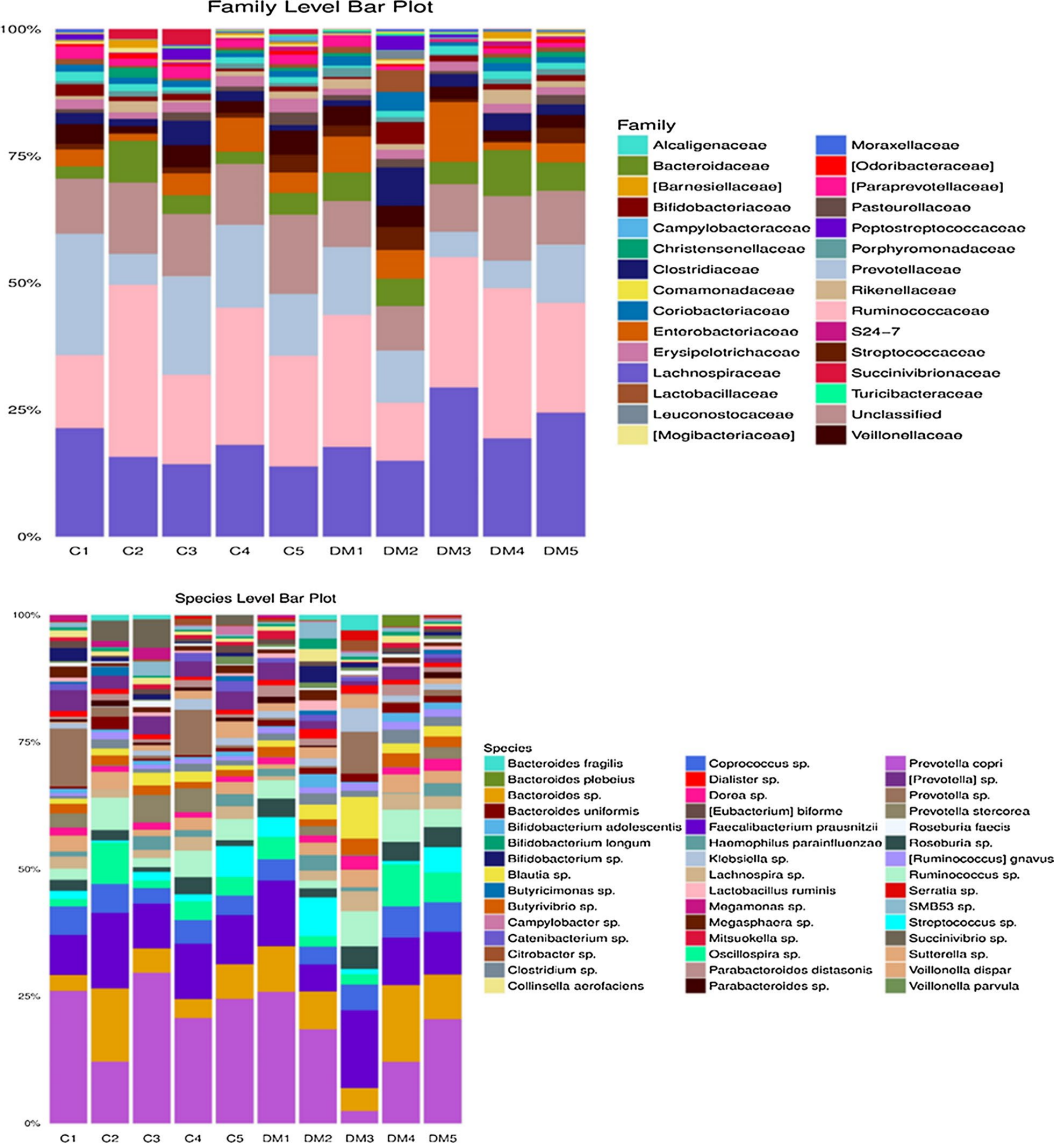
Stacked bar plots representing relative percentage of most abundant taxa in type 2 diabetic patients (N = 5) and healthy controls (N = 5). Stacked bar plots were generated using the normalized read count values

Among the subjects enrolled, 8 were male and 2 were female, the age ranged from 30 to 58 years. Participants with T2D were grouped based on HbA1c into three categories; 6.6–7.0% (n = 2), 7.1–8.5% (n = 2), and > 8.5% (n = 1); the average BMI was 30.536 (Additional file 1: Table S1). A total of 2,431,348 and 2,972,433 raw sequence reads were generated from control subjects and T2D subjects, respectively. A total of 1,248,393 and 1,544,225 rRNA sequences were identified in control subjects and T2D subjects, respectively. The average number of processed paired reads per sample was 292,773.6 reads for healthy controls and 359,210 reads for T2D subjects, respectively. The clustering of all the qualified sequences at the 97% similarity level generated a total of 8414 OTUs. From the healthy and T2D samples, 3887 (average 777.4 OTU per sample) and 4527 OTUs (average 905.4 OTU per sample) were detected, respectively (Additional file 1: Fig. S1; Table 1). The principal coordinate analysis plot of the unweighted UniFrac distance matrix distinguish DM2, DM3, and DM5 from the rest of the samples suggested the presence of different bacterial communities (Additional file 1: Fig. S2). At the phylum level, the common bacteria-Firmicutes, Bacteroidetes, Proteobacteria, and Actinobacteria were dominant among both T2D patients and healthy subjects. At the class level, most of the shared OTUs between T2D subjects and healthy subjects belonged to class Actinobacteria, Bacteroidia, Bacilli, Clostridia, Betaproteobacteria, and Gammaproteobacteria. The major bacterial families representing the gut microbiome profile of the subjects were Lachnospiraceae, Ruminococcaceae, Prevotellaceae, Bacteroidaceae, Enterobacteriaceae, and Bifidobacteriaceae. *Prevotella copri, Bacteroides sp., Faecalibacterium prausnitzii, Butyricimonas sp., Bacteroides fragilis, Oscillospira sp., Butyrivibrio sp., Dorea sp*. and *Dialister sp* among T2D subjects and healthy subjects (Fig. 1). According to G test (http://qiime.org/scripts/group_significance.html), *Prevotella copri, Lactobacillus ruminis, Lactobacillus vaginalis, Bifidobacterium adolescentis, Bifidobacterium longum, Bacteroides fragilis, Blautia producta, Dorea formicigenerans, Haemophilus parainfluenzae*, *Ruminococcus gnavus,* and *Bifidobacterium bifidum* were found in significantly higher abundance in T2D patients compared with healthy subjects. Of note, *Bacteroides uniformis, Faecalibacterium prausnitzii, Prevotella stercorea, Veillonella parvula, Veillonella dispar,* and *Roseburia faecis* had a significantly lower abundance in T2D patients compared with healthy subjects (Table 2).

**Table 1:** Read count statistics of control and T2D patients

Sample ID	Total paired end reads	Processed reads	Total identified rRNA sequences	Total OTUs Picked
C1	359,322	215,480	183,565	659
C2	528,285	314,104	264,448	1026
C3	379,927	236,256	209,914	600
C4	629,712	380,659	318,530	753
C5	534,102	317,369	271,936	849
DM1	524,791	312,518	269,809	754
DM2	633,448	392,035	333,970	703
DM3	360,215	224,639	202,970	894
DM4	344,695	193,327	150,162	801
DM5	1,109,284	673,531	587,314	1375

**Table 2:** Comparison of Bacterial genus/species abundance in type II diabetes and healthy individuals with statistically significant difference (P = 0.05)

Bacterial Genus/species	Mean abundance in Type 2 Diabetes	Mean abundance in Healthy Individuals	Status in Type 2 Diabetes of our Study	Phylum
*Prevotella copri*	5123.6	3048	Elevated	Bacteroidetes
*Lactobacillus ruminis*	14,550.4	340.8	Elevated	Firmicutes
*Lactobacillus vaginalis*	446.2	0.2	Elevated	Firmicutes
*Bifidobacterium adolescentis*	73.8	3.6	Elevated	Actinobacteria
*Bacteroides uniformis*	0.4	133.8	Reduced	Bacteroidetes
*Faecalibacterium prausnitzii*	0.4	67.8	Reduced	Firmicutes
*Bifidobacterium longum*	1718.8	228.2	Elevated	Actinobacteria
*Ruminococcus*	954.6	102.6	Elevated	Firmicutes
*Catenibacterium*	287.2	809	Reduced	Firmicutes
*Coprococcus*	1695.6	841.2	Elevated	Firmicutes
*Lachnospira*	984	221.8	Elevated	Firmicutes
*Enterobacter cloacae*	251	31.8	Elevated	Proteobacteria
*Butyrivibrio*	115.2	304.6	Reduced	Firmicutes
*Klebsiella*	2255.4	537	Elevated	Proteobacteria
*Haemophilus parainfluenzae*	592.4	17.8	Elevated	Proteobacteria
*Mitsuokella multacida*	432.2	175	Elevated	Firmicutes
*Prevotella stercorea*	0	409.8	Reduced	Bacteroidetes
*Bacteroides fragilis*	11,082	716.6	Elevated	Bacteroidetes
*Bifidobacterium bifidum*	98	0	Elevated	Actinobacteria
*Veillonella parvula*	91.2	332.8	Reduced	Firmicutes
*Hafnia alvei*	222.6	10.6	Elevated	Proteobacteria
*Butyricimonas*	99	0	Elevated	Bacteroidetes
*Bacteroides caccae*	296.6	10.2	Elevated	Bacteroidetes
*Veillonella dispar*	221.2	478.8	Reduced	Firmicutes
*Ruminococcus*	2.8	62.2	Reduced	Firmicutes
*Megasphaera*	21,795	822	Elevated	Firmicutes
*Collinsella aerofaciens*	492.4	326.8	Elevated	Actinobacteria
*Roseburia faecis*	43.8	111.2	Reduced	Firmicutes
*Dorea formicigenerans*	138.8	61.8	Elevated	Firmicutes
*Blautia producta*	159.6	84.8	Elevated	Firmicutes
*Citrobacter*	70.4	25.8	Elevated	Proteobacteria
*Ruminococcus gnavus*	14.4	0.2	Elevated	Firmicutes

## Discussion

Evidence suggest that T2D development could be associated with a high levels of pro-inflammatory cytokines, chemokines and inflammatory proteins in the peripheral circulation. Given that lipopolysaccharides (LPS) of gram-negative bacteria induce TLR-4 complex-mediated inflammatory responses that may drive the potential development of T2D [9], we also aimed to determine the signatures of inflammation and anti-inflammation associated with the observed gut microbiome profiles in T2D. *Lactobacillus spp., Bifidobacterium spp., Prevotella spp., Ruminococcus spp.,* and *Bacteroides spp.* generate acetate through the Wood–Ljungdahl and acetyl-CoA pathways. *Veillonella spp., Bacteroides spp., Megasphaera sp, Roseburia sp., Ruminococcus sp.,* and *Coprococcus sp* are responsible for the production of propionate through succinate, acrylate, and propanediol pathways. *Roseburia spp., Faecalibacterium prausnitzii,* and *Coprococcus sp* are responsible for the production of butyrate through the butyryl-CoA:acetate CoA-transferase routes and the phosphotransbutyrylase/butyrate kinase pathway. *Bacteroides fragilis, Lactobacillus, and Bifidobacterium* are involved in the metabolism of secondary bile acids. SCFAs induce secretion of cytokines by intestinal epithelial cells (IECs) through MEK–ERK and p38 MAPK pathways via activation of G-protein coupled receptors (GPRs) 41 and 43 [10]. Binding of SCFAs to G-protein coupled receptors (GPCRs) drive the activation of RAS, PKA, PI3K, ERK1/2, and ATF2 signalling cascade, which induce the secretion and release of inflammatory mediators such as IL-1, MCP1, IL-6, TNF-α, CXCL1, and CXCL2 [11]. This also activates the assembly of inflammasome and induces the secretion of Th1 polarising inflammatory cytokine, IL-18 [12] as well as IL-1β, both via activation of caspase 1. Firmicutes are gram-positive whereas Bacteroidetes are gram-negative bacteria. In obese individuals, a higher abundance of Firmicutes enhances the level of LPS that enhances the expression of inducible nitric oxide synthase (iNOS), which has been known to impair insulin sensitivity [13].

*Faecalibacterium prausnitzii* produces 15 kDa protein that drives anti-inflammatory milieu through the inhibition of NF-κB pathway in intestinal epithelial cells [14]. *Faecalibacterium prausnitzii* is considered to be the main butyrate producing bacteria in the intestine and prevents inflammation through the production of metabolites that maintain the function of intestinal barrier. Butyrate inhibits inflammation of intestinal mucosa through activation of PPARγ and inhibition of NF-κB and IFN-γ [15]. Butyrate induces hyperacetylation of histones through the prevention of histone deacetylase. *F. prausnitzii* augments anti-inflammatory activity through the expression of IL-10 in peripheral blood [16]. It is also involved in the fermentation of unabsorbed carbohydrate [17]. On the other hand, the lower abundance of *F. prausnitzii* reduces the level of butyrate production in the intestine. Butyrate induces the activation of free fatty acid receptor 2 that regulates the insulin signaling pathway in adipose tissue. It also induces the secretion of glucagon-like peptide 1 (GLP1) in the gut that prevents the accumulation of fat and enhances the sensitivity to insulin. Low level butyrate production due to lower abundance of *F. prausnitzii* induces the production of inflammatory cytokines through the activation of NF-κb [18].

*Lactobacillus ruminis* induces the secretion of proinflammatory cytokine IL-8 by gut epithelial cells and is also involved in the elevation of IL-6 in the serum of stroke patients [19]. An anaerobic gram-positive gut bacteria *Ruminococcus gnavus* is involved in the secretion of glucorhamnan polysaccharide with an L-rhamnose oligosaccharide backbone and glucose sidechains which drives the activation of TNFα in dendritic cells [20]. *Butyrivibrio* is a commensal bacteria involved in the production of butyrate through fermentation of pyruvate. Butyrate induces the secretion of an anti-inflammatory cytokine IL-10 from regulatory T cells (Treg). It also inhibits the proliferation of pathogenic bacteria in the gut by inducing secretion of IgA by plasma cells [21]. The abundance of *Butyricimonas* is associated with the production of inflammatory cytokines such as IL-1β and TGFβ1 in the ileum [22]. *C. aerofaciens* has genes that encode butyric acid kinase and phosphate butyryltransferase enzymes. It is involved in the biosynthesis of butyric acid which enhances inflammation in T2D [23]. OmpW protein produced by *Bacteroides caccae* induces an inflammatory response in clinical gout [24]. Bacteroides fragilis toxin (BFT) of *B. fragilis* induces activation of Stat3 that drive antimicrobial peptide secretion and glycosylation of mucus. LpxF of B. fragilis allows to bypassing the LPS induced TLR4 dependent phagocytic activity [25]. Reduced abundance of *B. uniformis* strains has been associated with intestinal inflammation [26]. Higher abundance of *Prevotella copri* and IL-6 showed positive association with the develop of T2D [27].

In conclusion, our findings showed that a higher abundance of inflammatory bacteria such as *Lactobacillus ruminis, Ruminococcus gnavus, Bacteroides caccae, Butyricimonas, Prevotella copri, and Collinsella aerofaciens* and lower abundance of anti-inflammatory bacteria such as *Faecalibacterium prausnitzii*, and *Butyrivibrio* may have a role in the development of T2D. The current small sample size based pilot metagenomic investigation provides a snap short of diagnostic markers and treatment targets in T2D patients.

## Limitations

This study was conducted with a limited number of T2D patients; thus, the gut bacterial signatures are required to validate in a larger cohort to understand the role of inflammatory and anti-inflammatory bacteria in T2D.

## Supplementary Information

**Additional file1: Figure S1**. Rarefaction plot analysis of V3 sequencing of 16S rRNA gene in faecal microbiota from T2D patients (DM1-DM5) and non-diabetic controls (C1–C5). **Figure S2**. Beta-diversity of the gut microbial communities in T2D patients and healthy controls. Principal Coordinates Analysis (PCoA) plot based on weighted (A) and unweighted (B) UniFrac distance. Each dot represents one sample from each group. **Table S1**. Clinical and demographic profile of type 2 diabetes mellitus subjects and healthy subjects.

## Data Availability

The datasets utilized in present study are available in supplementary file and raw data of sequencing are available from the corresponding author on reasonable request.

## Abbreviations

T2D: Type 2 diabetes
SCFAs: Short-chain fatty acids
OTU: Operational taxonomic unit
