# Supplementary material for: Could dysbiosis of inflammatory and anti-inflammatory gut bacteria have an implications in the development of type 2 diabetes? A pilot investigation
Source: BMC Res Notes. 2021 Feb 6;14:52. doi: 10.1186/s13104-021-05466-2 (PMC7868023; doi:10.1186/s13104-021-05466-2)
Supplement: Supplementary file 1 — Additional file1: Figure S1. Rarefaction plot analysis of V3 sequencing of 16S rRNA gene in faecal microbiota from T2D patients (DM1-DM5) and non-diabetic controls (C1–C5). Figure S2. Beta-diversity of the gut microbial communities in T2D patients and healthy controls. Principal Coordinates Analysis (PCoA) plot based on weighted (A) and unweighted (B) UniFrac distance. Each dot represents one sample from each group. Table S1. Clinical and demographic profile of type 2 diabetes mellitus subjects and healthy subjects. [file 13104_2021_5466_MOESM1_ESM.docx]

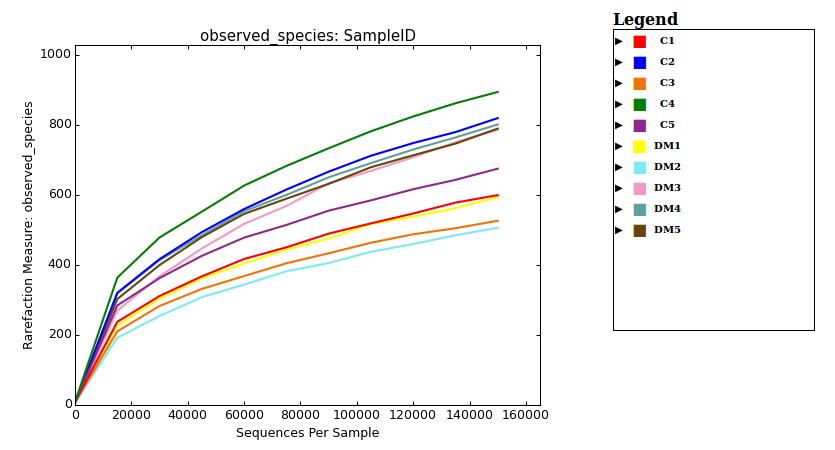


Supplementary Figure 1. Rarefaction plot analysis of V3 sequencing of 16S rRNA gene in faecal microbiota from T2D patients (DM1-DM5) and non-diabetic controls (C1–C5)

A


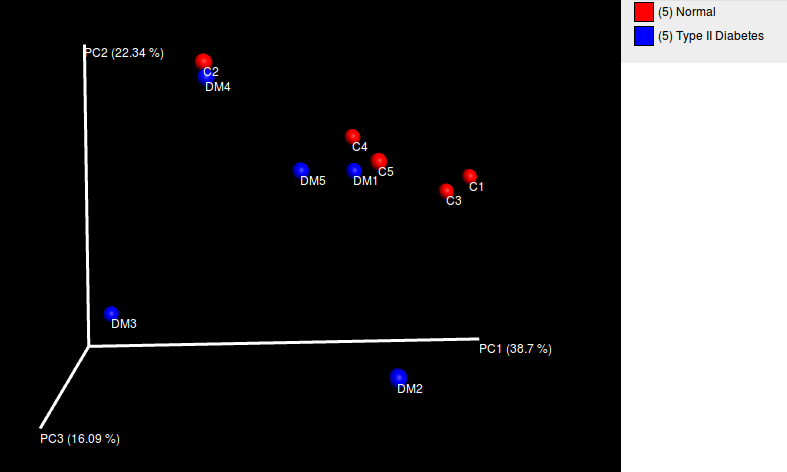


B


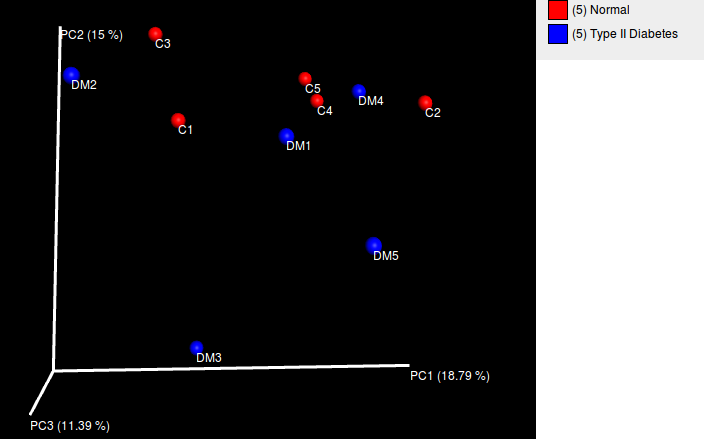


Supplementary Figure 2: Beta-diversity of the gut microbial communities in T2D patients and healthy controls. Principal Coordinates Analysis (PCoA) plot based on weighted (A) and unweighted (B) UniFrac distance. Each dot represents one sample from each group.

**Suppl. Table 1**: Clinical and demographic profile of type 2 diabetes mellitus subjects and healthy subjects:

| Subject ID | Age-ranges | Sex | FBS | PPBS | HbA1c (MBG) | BMI |
| --- | --- | --- | --- | --- | --- | --- |
| DM1 | 33-58 | F | 125 | 196 | 6.6(133.6) | 32.16 |
| DM2 |  | M | 118 | 191 | 6.9(152.6) | 31.36 |
| DM3 |  |  | 258 | 503 | 12(314) | 28.93 |
| DM4 |  |  | 137 | 239 | 7.9(184) | 25.33 |
| DM5 |  |  | 142 | 184 | 7.6(174) | 34.90 |
| C1 | 30-55 | M | 86 | 108 | 5.7(114) | 24.98 |
| C2 |  |  | 98 | 104 | 5.5(108) | 24.17 |
| C3 |  |  | 90 | 100 | 5.4(105.4) | 26.83 |
| C4 |  | F | 92 | 104 | 5.6(111) | 25.32 |
| C5 |  | M | 79 | 93 | 5.3(101) | 23.66 |

BMI: Body mass index (Kg/m^2^), HbA1C: Glycated hemoglobin (%), MBG: Mean Blood glucose; PPBS: Post Prandial Blood Sugar
